# Plant–environment interactions in Africa—Solutions to the challenges of environmental change

**DOI:** 10.1002/pei3.10063

**Published:** 2021-10-25

**Authors:** Wayne Dawson, Stacy Singer, Abdelbagi Ismail

**Affiliations:** ^1^ Department of Biosciences Durham University South Road, Durham DH1 3LE United Kingdom; ^2^ Agriculture and Agri‐Food Canada Lethbridge Research and Development Centre Lethbridge, AB, T1J 4B1 Canada; ^3^ International Rice Research Institute-Africa C/O ILRI Nairobi 30709 Kenya

Many aspects of environmental change represent a challenge to the plant sciences, from how to achieve sustainable crop production under climate change, to preserving plant diversity and the ecosystem services that plants provide in the face of land‐use change. The need to find solutions to the problems posed by environmental change is particularly strong in African countries. For this reason, we are dedicating a *P‐EI* Special Issue to this topic, which will be spread across two consecutive issues of the journal in 2022.

We are keen to promote the science of African researchers in the field of plant and environmental sciences in this Special Issue, and we are now seeking article submissions by researchers based in Africa for this Special Issue. In line with the Special Issue theme, we are particularly keen to consider articles that focus on the impacts that environmental change has on plant biodiversity and sustainable crop production, and on the potential solutions that will reduce those impacts. Articles can be from across the scope of the plant and environmental sciences that we normally consider for publication, including plant ecology and evolution, biodiversity, plant physiology and crop science, plant responses to biotic and abiotic stresses, and cell and molecular biology. We will also consider literature reviews, and shorter, more exploratory manuscripts under our “Interaction Insights” category. The research, the lead author and corresponding authors should be based in Africa. All accepted publications should meet our four key criteria (please see our author guidelines):

1. Does the subject of the study focus on our key criterion for publication: interactions between plants and their environment (abiotic or biotic)?
2. Is the study sound science? By that, we mean, are there no major flaws in the design, the logic or the analysis of the study, which would invalidate conclusions or inferences made? Do the methods and results actually address the main question/s or hypothesis/es of the study?
3. Does the study add knowledge to plant and environmental sciences? The study does not have to be 100% novel, but it also should not simply repeat what has been done before.
4. Is the quality of the manuscript writing and presentation sufficient for reviewers to focus on the quality and merit of the science in the study?

All manuscripts submitted for consideration in this Special Issue will be subject to initial screening and if deemed suitable, will be subject to peer review. Article publication charges will be waived for authors of accepted articles from low‐income, sub‐Saharan African countries—see the following link for more information: https://authorservices.wiley.com/open‐research/open‐access/for‐authors/waivers‐and‐discounts.html. All articles in this Special Issue will be published without cost to the contributing authors.

**The deadline for submitting manuscripts for in this Special Issue is the 28th of February 2022**. Please remember to select “yes” when asked if you are submitting a manuscript to be considered as part of the Special Issue.

Please email PEI@Wiley.com or the Editor‐In‐Chief (wayne.dawson@durham.ac.uk) if you would like to receive more details. We are excited to launch this Special Issue, and we look forward to receiving your submissions.

